# Ni(OH)_2_ nanosheets grown on porous hybrid g-C_3_N_4_/RGO network as high performance supercapacitor electrode

**DOI:** 10.1038/srep43413

**Published:** 2017-03-13

**Authors:** Lei Li, Jia Qin, Huiting Bi, Shili Gai, Fei He, Peng Gao, Yunlu Dai, Xitian Zhang, Dan Yang, Piaoping Yang

**Affiliations:** 1 Key Laboratory of Superlight Materials and Surface Technology, Ministry of Education, Harbin Engineering University, Harbin 150001, P. R. China; 2 Key Laboratory for Photonic and Electronic Bandgap Materials, Ministry of Education, School of Physics and Electronic Engineering, Harbin 150001, P.R. China

## Abstract

A porous hybrid g-C_3_N_4_/RGO (CNRG) material has been fabricated through a facile hydrothermal process with the help of glucose molecules, and serves as an efficient immobilization substrate to support ultrathin Ni(OH)_2_ nanosheets under an easy precipitation process. It was found that the g-C_3_N_4_ flakes can uniformly coat on both sides of the RGO, forming sandwich-type composites with a hierarchical structure. It is worth noting that the introduction of the g-C_3_N_4_ can effectively achieve the high dispersion and avoid the agglomeration of the nickel hydroxide, and significantly enhance the synthetically capacitive performance. Owning to this unique combination and structure, the CNRG/Ni(OH)_2_ composite possesses large surface area with suitable pore size distribution, which can effectively accommodate the electrolyte ions migration and accelerate efficient electron transport. When used as electrode for supercapacitor, the hybrid material exhibits high supercapacitive performance, such as an admirable specific capacitance (1785 F/g at a current density of 2 A/g), desirable rate stability (retain 910 F/g at 20 A/g) and favorable cycling durability (maintaining 71.3% capacity after 5000 cycles at 3 A/g). Such desirable properties signify that the CNRG/Ni(OH)_2_ composites can be a promising electrode material in the application of the supercapacitor.

Supercapacitors, as an essential part of applicable energy storage devices, have been widely employed in energy backup systems, electrical vehicles and portable electronic devices owing to their fast recharge ability, high power density, and environmental friendliness^1^,^2^,^3^. Generally, supercapacitors can be divided into two categories according to the energy storage mechanism: electrical double-layer capacitors (EDLCs) that store energy depending on the pure electrostatic charge accumulated at the interface of electrode/electrolyte, such as carbon materials with high specific area and excellent conductivity^4^,^5^,^6^,^7^,^8^, and pseudocapacitors whose capacitance originates from reversible Faradaic reaction in the presence of electro-active species in the electrodes, including conducting polymers, transition metal oxides and hydroxides^9^,^10^,^11^. Pseudocapacitors exhibit much better capacitive behavior due to the fast and reversible faradic reaction compared to EDLCs^12^,^13^,^14^,^15^. However, during the long-term Faradaic processes, undesirable high rate stability and reversibility for these electrode materials have been critical drawbacks that hinder their practical applications^16^,^17^,^18^,^19^. Therefore, the constructing of new structured materials which integrated these two charge storage mechanisms, can take fully utilization of their synergistic mechanism and attain the goal of gaining eligible capacitor electrode^20^,^21^,^22^.

Among all sorts of pseudo-active materials, nickel hydroxide is regarded as one of the most favorable material for capacitor electrode owning to its easy preparation, low cost and favorable theoretical specific capacitance^23^,^24^,^25^,^26^. Nevertheless, both poor electronic conductivity and large volumetric expansion of nickel hydroxide significantly restrict the electron transport and decelerate the redox reactions^27^,^28^,^29^. Moreover, it is subjected to aggregation upon cycling and unstable electrochemical interface between active material and electrolyte ascribed to an excessive surface energy^30^,^31^. To address this problem, one feasible and effective approach is to design novel hybrid structure, which can optimize this pseudocapacitive materials to nano-size (nanoparticles or nanosheets) and confine them within a conductive carbonaceous matrix^32^,^33^,^34^,^35^.

Graphene, a new member of carbonaceous material, can represent a desirable template in dispersing or wrapping active nanoparticles or nanosheets, avoiding aggregation and facilitating charge transportation benefiting from its high specific surface and extraordinary conductivity^36^,^37^,^38^. In particular, its two-dimensional sp^2^-bonded structure can also provide great opportunity to increase the cycling stability of the as-synthesized material for the use of supercapacitor electrode^39^. With regard to the synthesis of graphene and Ni(OH)_2_ hybrid material, the direct formation of nano-sized Ni(OH)_2_ is inadvisable because of the weak bonding between nickel hydroxide and graphene leading to the falling of active nanoparticles or nanosheets away from the substrate^40^. To solve this problem, graphitic carbon nitride (g-C_3_N_4_) can be emerging as a suitable candidate combined with graphene for high loading nanocomposites^41^. For one thing, owning to the porous structure and high nitrogen content, g-C_3_N_4_ can offer more highly reactive region and binding defects to serve as an eligible template for crystals’ nucleating and growing^42^,^43^,^44^. Additionally, g-C_3_N_4_ has lamellar structure and good lattice which can be well matched with graphene and hydroxide nanosheets^45^,^46^. For another thing, it was found that the combination of g-C_3_N_4_ with graphene can also enhance the electrochemical performance of g-C_3_N_4_^47^. Thus, it is of great significance to combine pseudocapacitive materials with carbon materials for high-performance electrode with the help of g-C_3_N_4_ nanosheets.

In this work, we construct a balanced and porous hierarchical CNRG/Ni(OH)_2_ architecture, which can take advantage of the desirable conductivity of the graphene and trigger a synergistic effect from EDLCs and Ni(OH)_2_ nanosheets. By utilizing g-C_3_N_4_ as template, abundant ultrathin Ni(OH)_2_ nanosheets anchored on the carbon-based material firmly without agglomeration. Such unique structure significantly increases ionic accessibility and transmission, thus accelerating the rapid diffusion of electrolyte to access the active sites of Ni(OH)_2_ nanosheets. When employed as electrode in supercapacitor, the CNRG/Ni(OH)_2_ exhibits excellent electro-chemical performance in terms of specific capacitance, rate capability and cycling performance in comparison with pure Ni(OH)_2_. On account of the remarkable properties of the as-prepared samples, we infer that this novel route towards the synthesis of electrode material will have practical application in the fields of conversion and energy storage systems.

## Results and Discussion

### Phase and Morphology Properties

**Figure 1:**
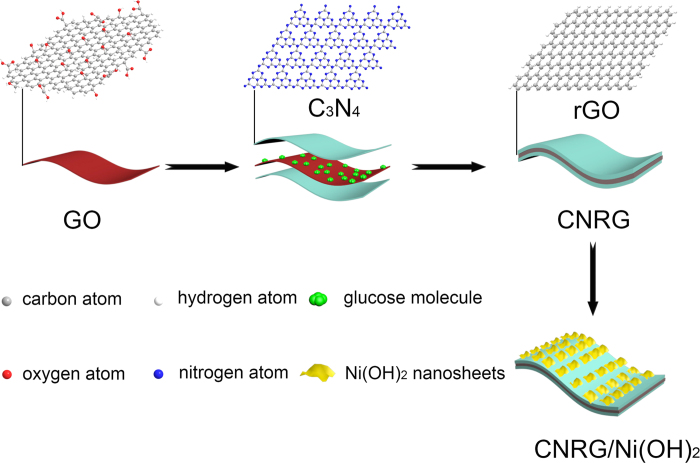
Schematic illustration for the preparation of CNRG/Ni(OH)_2_ composite.

Figure 1 illustrates the overall mechanism for the preparation of CNRG/Ni(OH)_2_ *via* two main procedures. Firstly, the synthesis of CNRG was finished through a hydrothermal treatment with the aid of ammonia and glucose. In this process, oxygen-containing groups on GO sheets can be favorably bonded with glucose molecule via electrostatic and hydrogen conjugating interactions, which can not only serve as binder for the g-C_3_N_4_ but also reduce GO to RGO with the help of ammonia hydroxide. Afterwards, with the addition of Ni(Ac)_2_, Ni^2+^.ions can be bound onto the interior surface of g-C_3_N_4_, because plentiful nitrogen pots on the surface of g-C_3_N_4_ can generate strong chemical absorption with metal ions. Owning to this desirable effect, ultrathin Ni(OH)_2_ nanosheets can uniformly deposit on the surface of C_3_N_4_ layer by a facile chemical precipitation method, triggering the formation of ternary g-C_3_N_4_/RGO/Ni(OH)_2_ composites with increased hierarchy.

**Figure 2:**
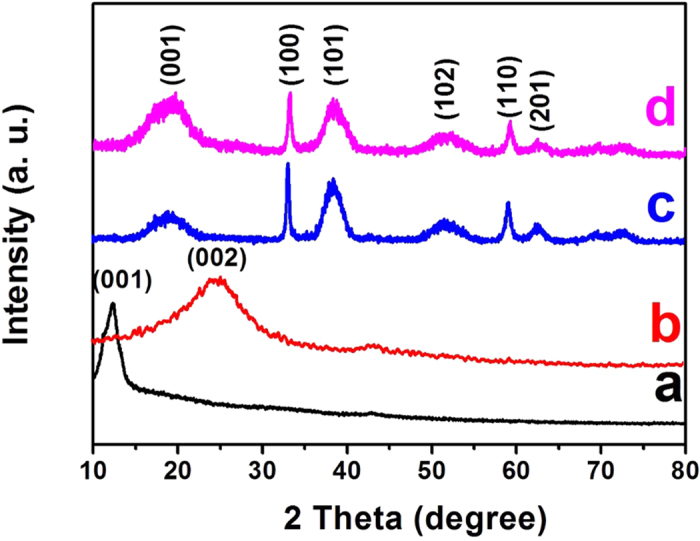
XRD patterns of GO (**a**), CNRG (**b**), pure Ni(OH)_2_ (**c**) and CNRG/Ni(OH)_2_ composite (**d**).

XRD patterns of as-synthesized GO, CNRG, pure Ni(OH)_2_ and CNRG/Ni(OH)_2_ are shown in Fig. 2. The sharp and intense diffraction peak of GO locates at about 11.6° corresponds to a layered structure, which is calculated to be a basal spacing of 0.76 nm (larger than that of previous graphite (0.34 nm)), indicating that oxygen groups are introduced into the graphitic layer and a well ordered structure of GO has emerged^22^. After the GO is reduced by glucose and coated with C_3_N_4_ nanosheets under hydrothermal process, we can see that the peak at 11.6° disappears and a broad peak at about 24.7° can be observed with interlayer spacing of 0.359 nm for CNRG, revealing that most of the oxygen functional groups of GO have been removed, which is beneficial to a further increasing in electrical conductivity^24^,^48^. For pure Ni(OH)_2_ sample, it can be seen that all the peaks of the XRD patterns can be well indexed to the β-Ni(OH)_2_ (JCPDS No. 14−0117). CNRG/Ni(OH)_2_ has the similar diffraction pattern compared to that of pure Ni(OH)_2_, implying that Ni(OH)_2_ nanosheets can be well formed on the surface of C_3_N_4_. It is noteworthy that no characteristic peaks of GO or RGO can be found in the patterns of CNRG/Ni(OH)_2_, demonstrating that high crystalline of Ni(OH)_2_, which can be further confirmed by following characterization.

**Figure 3:**
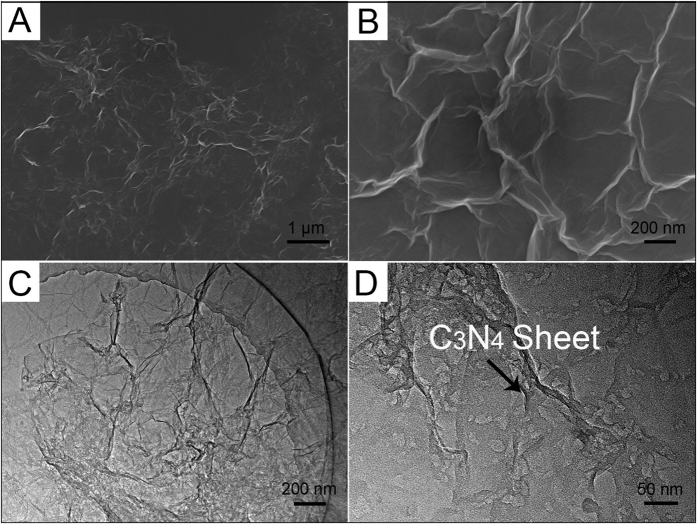
SEM (**A**,**B**) and TEM (**C**,**D**) images of CNRG composite.

FESEM and TEM were employed to characterize the morphology and structure of CNRG. As displayed in Fig. 3A and B, the CNRG exhibits a silk-like morphology with a lot of winkles, which could prevent the sheets from stacking on each other. The rather smooth surface of CNRG confirms that the g-C_3_N_4_ have uniformly attached to GO substrate without any obvious agglomeration. The TEM images (Fig. 3C and D) of CNRG clearly show the uniformly dispersion of C_3_N_4_ nanosheets onto the surface of RGO. As presented in Fig. 3D, the resulting macroporosity of the C_3_N_4_ can serve as basal plane for high mass loading of Ni(OH)_2_ and facilitate charge transport at high current density.

**Figure 4:**
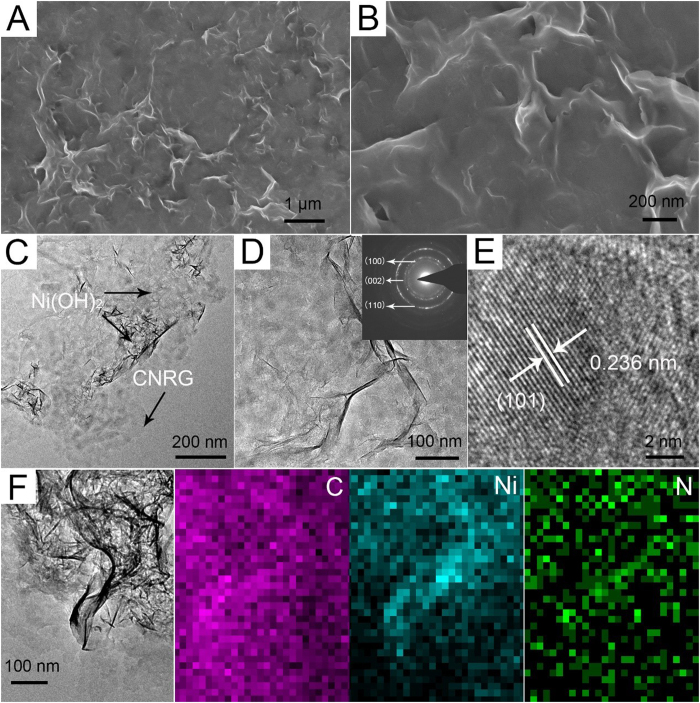
Low- and high-magnified SEM image (**A** and **B**), TEM images (**C** and **D**), SAED (inset of **D**), HRTEM image (**E**), HAADF-STEM image and elemental mapping images (**F**) of CNRG/Ni(OH)_2_ composite.

Figure 4A and B show the SEM images of the CNRG/Ni(OH)_2_ composites, which exhibit a relatively rough surface compared with CNRG due to the adhesion of Ni(OH)_2_ nanopetals. In addition, as displayed in Figure S1, the hybrid electrode can also maintain the morphology of the materials. From the TEM images of the CNRG/Ni(OH)_2_ composites (Fig. 4C and D), we can clearly observe that the surface of CNRG is covered by ultrathin Ni(OH)_2_ nanosheet densely. Owning to the strong interaction between g-C_3_N_4_ and Ni(OH)_2_, layered Ni(OH)_2_ nanosheets are firmly immobilized on surface of the porous CNRG and not peel off even after a long period of vigorous ultrasonic treatment for the preparation of TEM specimen. The selected area electron diffraction (SAED) pattern (inset of Fig. 4D) further shows the growth of Ni(OH)_2_ nanocrystal on CNRG *in situ*. And the HRTEM image in Fig. 4E reveals that the calculated lattice spacing of the nanosheets is about 0.236 nm, which corresponds to the (101) plane of β-Ni(OH)_2_. EDS mapping technique was conducted to determine the compositional and elemental distribution, and the results are shown in Fig. 4F. It can be clearly seen that the elements of C, Ni, and N are homogenously distributed, demonstrating the presence of C_3_N_4_ nanosheets and the uniform coating of Ni(OH)_2_ nanosheets. Such unique ternary structure can provide more electrochemically active sites for Ni(OH)_2_ to be exposed by electrolyte, meanwhile favor the ion transfer and diffusion, thus accelerating the surface redox reaction.

**Figure 5:**
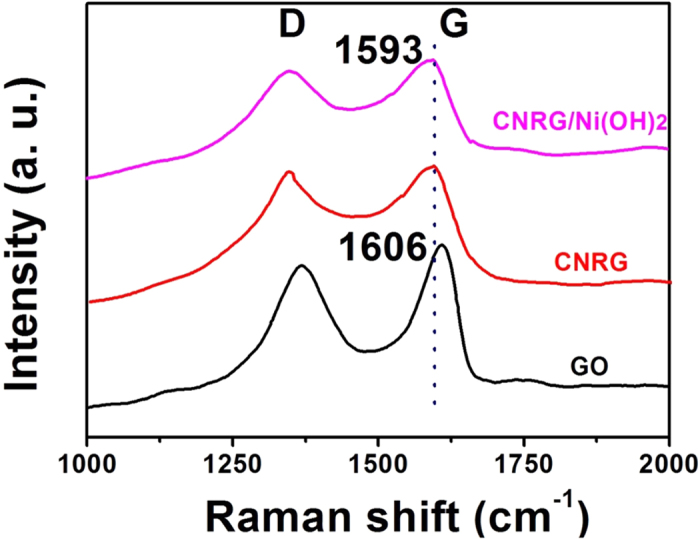
Raman spectra of GO, CNRG and CNRG/Ni(OH)_2_ composite.

Raman spectra have been employed to clarify the degree of graphitization and the effect of nitrogen doping of RGO. Figure 5 presents the Raman spectrums for GO, CNRG and CNRG/Ni(OH)_2_. As known, the intensity ratio of D band versus G band (I_D_/I_G_) is a significant parameter to the carbon hybridization state of materials and the degree of disorder^48^,^49^. An increased value (I_D_/I_G_) is obtained from GO (0.923) to those of CNRG (0.983) and CNRG/Ni(OH)_2_ (0.981), implying the removal of the oxygen functional groups occurred on GO and more defects appear due to the heteroatomic doping of N from C_3_N_4_ nanosheets. Noteworthy, the G band of CNRG presents a downshift compared with that of GO, which is also caused by the nitrogen doping into the RGO framework^50^,^51^.

**Figure 6:**
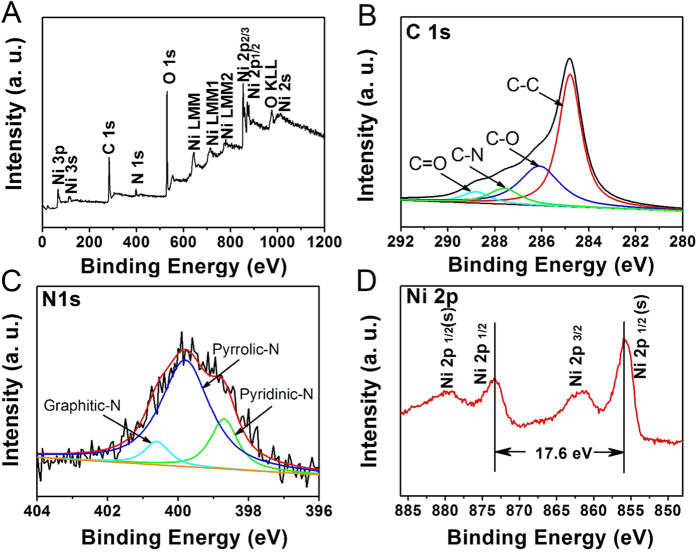
XPS spectra of CNRG/Ni(OH)_2_ composite: survey spectrum (**A**), C 1 s (**B**), N 1 s (**C**) and Ni 2p (**D**).

The elemental composition and chemical valence for CNRG/Ni(OH)_2_ composite were also elucidated by X-ray photoelectron spectra analysis. The survey spectrum (Fig. 6A) reveals the coexistence of C, Ni, O and N elements, which is consistent with the results of EDS mapping. And the high-resolution of C 1 s spectrum (Fig. 6B) can be fitted into a dominant peak and three relatively weak peaks, which are respectively assigned to sp^2^ carbon (284.8 eV), C-O (286.1 eV), C-N (287.9 eV) and carbon in carbonyl (288.8 eV). This result suggests that during the hydrothermal process, the oxygen functional groups have been partially removed and the C_3_N_4_ nanosheets have been successfully introduced onto RGO^30^,^52^. Figure 6C depicts the high XPS resolution spectra of N 1 s on CNRG/Ni(OH)_2_. Deconvolution of the core-level N 1 s shows three peaks at 398.7, 399.5 and 400.4 eV, corresponding to three different types of nitrogen states graphitic N, pyrrolic N and pyridinic N, which are consistent with the characteristic nitrogen species of the g-C_3_N_4_^44^,^47^. Previous reports have confirmed that the graphitic N can be conductive to improve the electrical conductivity of carbon-based electrode, and pyrrolic N and pyridinic N are able to create plenty of active sites and extrinsic defects, which are helpful for the fast transportation of the ions and increase capacitance of the composites^4^,^53^. In addition, the peaks located at 879.6 eV and 861.1 eV can be assigned to Ni 2p_1/2_ and Ni 2p_3/2_ satellites, respectively, with a spin-energy separation of 17.6 eV, which is characteristic of the Ni(OH)_2_ phase^54^,^55^.

**Figure 7:**
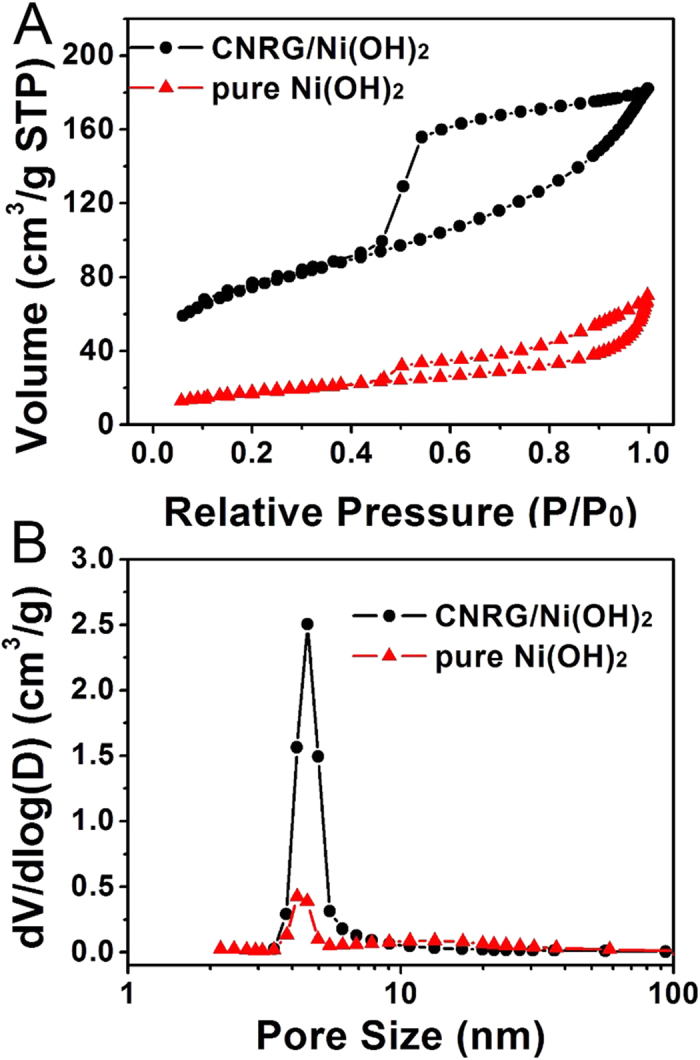
N_2_ adsorption/desorption isotherms (**A**) and the corresponding pore size distributions (**B**) of pure Ni(OH)_2_ and CNRG/Ni(OH)_2_ composite.

It is well accepted that surface area and pore size are two crucial factors to determine the properties of electrode materials^56^,^57^. Figure 7A displays the nitrogen adsorption and desorption isotherms for CNRG/Ni(OH)_2_ composite and pure Ni(OH)_2_, and both of which exhibits a typical type IV with a H_3_ hysteresis loop, suggesting the existence of mesopores of each sample. And the BET specific surface area of CNRG/Ni(OH)_2_ is measured to be 250 m^2^/g, which is markedly larger than that (63 m^2^/g) of pure Ni(OH)_2_, suggesting that positive effect of CNRG to avoid the aggregation of Ni(OH)_2_, leading to higher exposure of the active sites. In comparison with pure Ni(OH)_2_, the pore-size distribution curve of CNRG/Ni(OH)_2_ shows narrow size distribution (4.1 nm) and a prominent volume increase in the range of 3–5 nm, which is favorable for electrochemical reactions^58^,^59^. Such unique mesoporous structure of CNRG/Ni(OH)_2_ can not only guarantee a large electrode/electrolyte interface for electrostatic charge accumulation but also facilitate ion transport by shortening diffusion pathway, which is beneficial to the performance of the supercapacitor.

### Electrochemical properties

**Figure 8:**
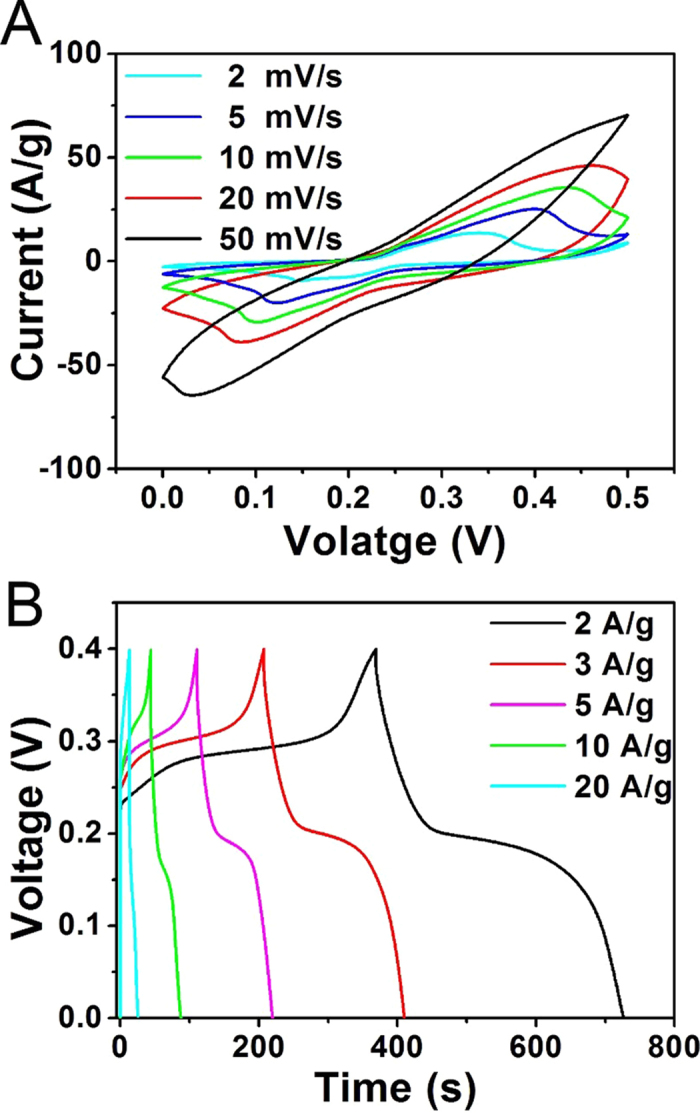
Cyclic voltammograms (**A**) of CNRG/Ni(OH)_2_ electrodes measured at scan rates from 2–50 mV/s, and charge-discharge curves (**B**) of CNRG/Ni(OH)_2_ measured at various discharge current.

**Figure 9:**
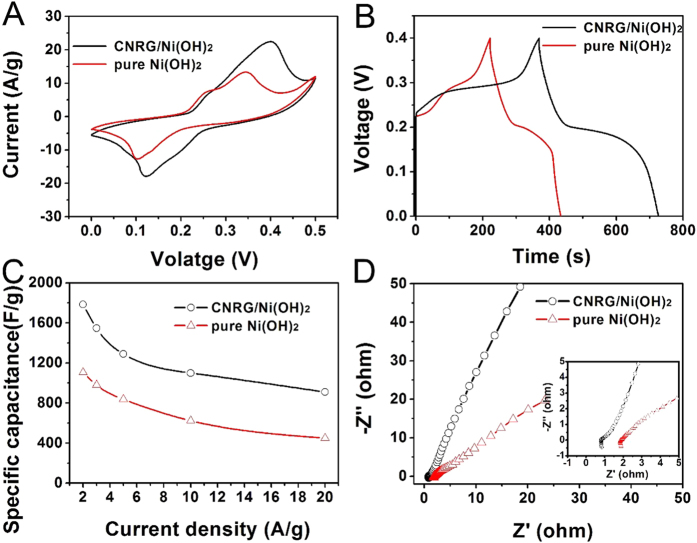
Cyclic voltammograms (CVs) curves (**A**), galvanostatic (GV) charge-discharge curves (**B**), current density dependence of the specific capacitance (**C**), and Nyquist plots of the EIS for CNRG and CNRG/Ni(OH)_2_ composite (**D**).

To determine the potential application of CNRG/Ni(OH)_2_ composite for high-performance supercapacitor, the cyclic voltammetry (CV) tests were carried out at various scan rates within the potential range from 0 to 0.5 V. As displayed in Fig. 8A, the potential of oxidation and reduction peaks shift towards more positive and negative direction with the increasing of the scan rates, which can be ascribed to the high electric polarization during the faradaic redox reaction at high scan rates. The redox peaks exhibit a symmetric shape, manifesting a high reversibility of this hybrid electrode materials. Moreover, the CV curves of CNRG/Ni(OH)_2_ maintain a relatively similar shape at each scan rates, indicating that the electrode possesses a desirable rate stability owning to the good adsorption and facile ion diffusion properties^60^. To explore the capacitive characteristics of this composite, the charging-discharging tests were operated and the plots of voltage versus time at various densities were displayed in Fig. 8B. It can be observed that potential plateaus are presented in charge-discharge curves which match well with the peaks of the CV curves, implying the pseudo-capacitive behavior of CNRG/Ni(OH)_2_. And the discharge time decreases monotonically with the increasing of current densities, due to drastic redox reaction to satisfy fast potential change. And the value of specific capacitance for the composite has been calculated and the corresponding data is displayed in Fig. 9C for comparison.

To determine the positive role of CNRG on the improvement of supercapacitive properties for nickel hydroxide, the CV curves for CNRG/Ni(OH)_2_ and pure Ni(OH)_2_ at the scan rate of 5 mV/s are shown in Fig. 9A. It can be clearly seen that a couple of highly reversible redox peaks emerge in each curve during the cathodic and anodic sweeps, which can be ascribed to the redox reaction between Ni(OH)_2_ and NiOOH in alkaline solution as follows: Ni(OH)_2_ + OH– ↔ NiOOH + H_2_O + e^−^ 35,61. Generally, the integral area of the CV curve is proportional to the value of capacitance^34^. And it can be figured out that the CV curve of CNRG/Ni(OH)_2_ owns much larger area than that of pure Ni(OH)_2_, suggesting that greatly enhanced specific capacitance has been obtained due to the introduction of CNRG. And the charge-discharge tests have been also conducted to verify better electrochemical performance of CNRG/Ni(OH)_2_. In Fig. 9B, the curve of CNRG/Ni(OH)_2_ composite presents a much longer discharge time than that of pure Ni(OH)_2_, which further confirm the much favorable specific capacitance of CNRG/Ni(OH)_2_. Figure 9C exhibits the rate performance of as-prepared samples according to the capacitive value versus different current densities. Encouragingly, CNRG/Ni(OH)_2_ composite possesses much higher capacitive performance than that of pure Ni(OH)_2_ at each current density. And we have included the total mass of Ni(OH)_2_ and CNRG to determine specific capacitance of the CNRG/Ni(OH)_2_ composite. Based on the Equation 1, the specific capacitance for CNRG/Ni(OH)_2_ at 2 A/g can be calculated as 1785 F/g, and retain at 910 F/g with the current density up to 20 A/g, demonstrating desirable rate capability, which is an important factor for the electrode materials to provide high power density^8^,^13^. Whereas at the same density, pure Ni(OH)_2_ exhibits comparatively poor specific capacitance of 1106 F/g and 450 F/g with an unsatisfied retention of 41%. The reason for the admirable specific capacitance and rate performance of this hybrid electrode is the interactive effect between the CNRG and Ni(OH)_2_. On one hand, ultra-thin Ni(OH)_2_ nanosheets can offer amount of active sites for faradaic reaction and account for dominant electrochemical capacitance; on the other hand, the CNRG can avoid the agglomeration of Ni(OH)_2_ nanosheets and enhance ion transfer.

The remarkably electrochemical performance for the CNRG/Ni(OH)_2_ hybrid electrode was further assessed through the electrochemical impedance spectroscopy measurements within the frequency range from 0.1 Hz to 100 kHz. As shown in Fig. 9D, the impedance plots of both samples consists of a semicircle in the high-frequency region and a relative straight line in the low-frequency region. As generally accepted to us, the semicircle diameter of EIS curve represents the electrochemical reaction impedance of the electrode, and the straight line is associated with the ion-diffusion resistance^20^,^62^. As presented in inset of Fig. 9D, the CNRG/Ni(OH)_2_ displays a much smaller semicircle over the high frequency range, and a more upright line than those of pure Ni(OH)_2_, implying this hybrid composite possesses faster ion diffusion process and lower charge transfer resistance during the faradic reaction^63^. It can be concluded that the high electronic conductivity of the CNRG/Ni(OH)_2_ can be ascribed to the unique structure of CNRG with large surface area and porous feature, which can make Ni(OH)_2_ nanosheets keep highly interconnected with each other to facilitate the electron transport.

**Figure 10:**
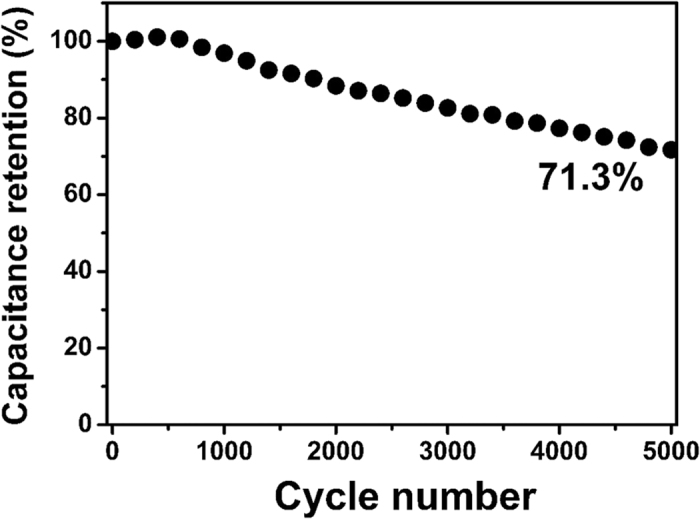
Cycling performance of CNRG/Ni(OH)_2_ composite measured at a current density of 3 A/g.

As cycling performance is a decisive parameter for applications of supercapacitor electrode, the stability test of CNRG/Ni(OH)_2_ composite was conducted *via* charge-discharge technique at 3 A/g for 5000 cycles. As shown in Fig. 10, for the cycling performance, during the initial cycles, the capacitance of CNRG/Ni(OH)_2_ presents a slight increase, which can be ascribed to electrode activation ascribed to the increasing of available active sites and the gradual diffusion of the trapped ions during activation process. Notably, the capacitance of CNRG/Ni(OH)_2_ composite retains 71.3% after 5000 charge-discharge cycles, suggesting that this electrode exhibits much admirable cycling electrochemical durability under identical test conditions. This result indicates that the synergistic combination between CNRG and Ni(OH)_2_ can effectively prevent aggregation of the active materials and accommodate the volume change during cycling process.

Above all, this work reports a novel and facile design for the fabrication of the g-C_3_N_4_/RGO (CNGR) mesoporous hybrid framework to serve as substrate for the formation of the Ni(OH)_2_ nanosheets. Because of the localized highly reactive region and binding defects of the g-C_3_N_4_, the CNRG can offer a large number of anchoring sites and prevent the agglomeration of the Ni(OH)_2_. Benefiting from the rational structural features which can effectively favor the ion transfer and diffusion, this composite exhibits excellent specific capacitance, desirable rate capability and cycling durability when served as electrode. These results demonstrate that the CNRG/Ni(OH)_2_ material with unique structure can be a promising electrode material for supercapacitor application.

## Methods

### Synthesis of g-C_3_N_4_/RGO (CNRG) composite

Graphene oxide (GO) was synthesized based on a modified Hummers method through the oxidation of natural graphite powder^22^. For the preparation of g-C_3_N_4_, melamine as precursor was calcinated for 3 h in the air atmosphere under 550 °C with a rate ramp of 4 °C min^–1^. Then a certain amount bulk g-C_3_N_4_ was decentralized uniformly in 30 mL of distilled water with ultra-sonication for over 18 h. For the synthesis of CNRG, 25 mL of 2.8 mg mL^–1^ GO solution was dispersed in the solution of g-C_3_N_4_, added with 0.5 g glucose and 1 mL ammonia, and then moved into a high pressure autoclave to keep for 12 hours at 180 °C. When the reaction was cooled to room temperature naturally, the obtained precipitates were centrifuged and washed with deionized water for several times.

### Synthesis of CNRG/Ni(OH)_2_ composites

The above products with the addition of 4 mL ammonia were dissolved in 170 mL of solvent (water/ethanol = 1/1, *V/V*) by hyperacoustic treatment. And then the solution was transferred into a three-necked flask under continuous stirring. After 20 min, 2.5 mmol of Ni(Ac)_2_ was gradually added into the above homogeneous solution and refluxed at 85 °C in an oil bath for 6 h. The products were collected by centrifugation and washed with distilled water and ethanol three times respectively. Finally, the precipitation was dried in a vacuum oven at 60 °C for 12 h. Pure Ni(OH)_2_ was prepared in the same method in the absence of CNRG composites as a comparison.

### Fabrication of electrode and electrochemical measurement

The electrochemical properties of CNRG/Ni(OH)_2_ are evaluated by previous method. The conventional three-electrode cell was consisted of the counter electrode (Pt foil of 1 × 1 cm^2^), the reference electrode (a Ag/AgCl electrode) and working electrode (Ni foam coated with active material), respectively. The weight of the active materials is about 3.5 mg. All measurements were conducted at room temperature and the electrolyte is the 6 M KOH aqueous solution. According to galvanostatic charge-discharge curves, the specific capacitance values of the electrode can be calculated by the following equation: C = IΔt/mΔV (1), where I is the response current density, Δt is the discharge time, m is the mass of the active materials on single electrode, ΔV is the potential range during the charge-discharge measurement.

### Characterization

Crystalline structure, the morphology, and chemical composition of the samples were investigated by powder X-ray diffraction (XRD) (Rigaku D/max TTR-III diffractometer with graphite monochromatized Cu Kα radiation (λ = 0.15405 nm)), scanning electron microscope (SEM, JSM-6480A), transmission electron microscopy (TEM, FEI Tecnai G2 S-Twin), high-resolution transmission electron microscopy (HRTEM), and the X-ray photoelectron spectra XPS (VG ESCALAB MK II electron energy spectrometer using Mg KR (1253.6 eV) as the X-ray excitation source). Raman spectra were conducted on a confocal laser microRaman spectrometer (LABRAM-HR, JY Co.), and N_2_ adsorption/desorption isotherms were measured from Micromeritics ASAP Tristar II 3020 apparatus. The electrochemical properties were carried out by a CHI 666D electrochemical workstation. All the tests were carried out at room temperature.

## Additional Information

**How to cite this article:** Li, L. *et al*. Ni(OH)_2_ nanosheets grown on porous hybrid g-C_3_N_4_/RGO network as high performance supercapacitor electrode. *Sci. Rep.* **7**, 43413; doi: 10.1038/srep43413 (2017).

**Publisher's note:** Springer Nature remains neutral with regard to jurisdictional claims in published maps and institutional affiliations.

## Supplementary Material

Supplementary Information
