# Supplementary material for: Ni(OH)2 nanosheets grown on porous hybrid g-C3N4/RGO network as high performance supercapacitor electrode
Source: Sci Rep. 2017 Mar 13;7:43413. doi: 10.1038/srep43413 (PMC5347133; doi:10.1038/srep43413)
Supplement: Supplementary Information [file srep43413-s1.doc]

**Supplementary Information**

**Ni(OH)_2_ nanosheets grown on porous hybrid g-C_3_N_4_/RGO network as high performance supercapacitor electrode**

**Lei Li,^1^** **Jia Qin,^1^ Huiting Bi,^1^ Shili Gai,^1^ Fei He^1^, Peng Gao^1, *^, Yunlu Dai ^1^, Xitian Zhang^2, *^, Dan Yang^1^, and Piaoping Yang^1, *^**

**
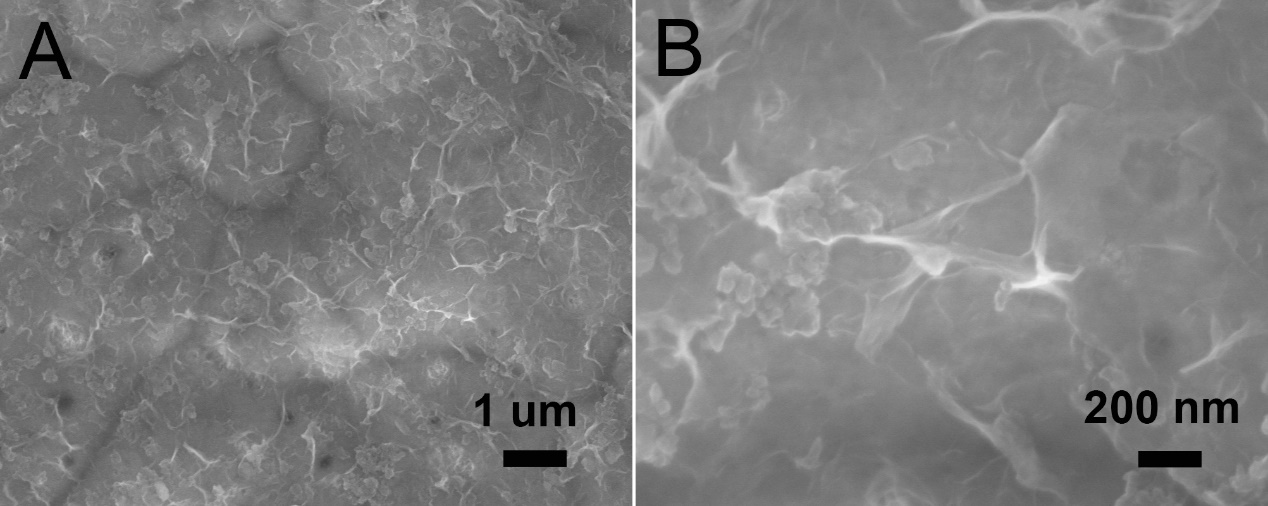
**

**Figure. S1** The SEM image (A and B) of hybrid electrode
